# Co-creation of HIVST delivery approaches for improving urban men’s engagement with HIV services in eThekwini District, KwaZulu-Natal: nominal group technique in intervention development

**DOI:** 10.1186/s40814-022-01083-3

**Published:** 2022-06-09

**Authors:** Tivani Mashamba-Thompson, Richard Lessells, Tafadzwa Dzinamarira, Paul Drain, Lehana Thabane

**Affiliations:** 1 Faculty of Health Science, University of Pretoria, Pretoria, South Africa; 2 CIHR Canadian HIV Trials Network, Vancouver, BC V6Z 1Y6 Canada; 3 KRISP, 1st Floor, Doris Duke Medical Research Institute, 719 Umbilo Road, Durban, 4041 South Africa; 4 School of Health Systems & Public Health, University of Pretoria, Pretoria, 0002 South Africa; 5 International Clinical Research Center, Department of Global Health, University of Washington, Seattle, USA; 6 Division of Infectious Diseases, Department of Medicine, University of Washington, Seattle, USA; 7 Department of Epidemiology, University of Washington, Seattle, USA; 8 Department of Health Research Methods, Evidence, and Impact, McMaster University, Hamilton, ON Canada; 9 Biostatistics Unit, Research Institute at St Joe’s Hamilton, St. Joseph’s Healthcare-Hamilton, Hamilton, ON Canada

**Keywords:** Men, Urban, HIV self-testing, Delivery

## Abstract

**Background:**

HIV self-testing (HIVST) is one of the recommended approaches for HIV testing services, particularly for helping reach populations who would not normally access facility-based HIV testing. Key stakeholder engagement is paramount in tailoring health interventions to ensure uptake by target populations.

**Objective:**

The main objective of this study was to collaborate with key stakeholder in the co-creation of an acceptable HIVST delivery strategies to help improve urban men’s engagement with HIV services.

**Methods:**

We invited key stakeholders for urban men’s HIV services to participate in a co-creation workshop aimed at developing HIVST delivery approaches for urban men, using eThekwini municipality as a study setting. We conducted purposive sampling to include health care users and health care providers, representing a range of views across the public sector and voluntary sector. We employed the nominal group technique (NGT) method for data collection. The NGT workshop was conducted in two consecutives: phase 1 was focused on determining barriers for men’s engagement with the current/facility-based HIV testing services; phase 2 was aimed at determining HIVST delivery strategies.

**Results:**

Participants identified the following factors as the most important barriers to uptake of HIV testing services by urban men: stigma, ignorance about the importance of testing, and testing process as well as fear of positive test results. Key stakeholders suggested internal motivation strategies as a potentially effective approach to support HIVST delivery strategy. Guided by the NGT results, we designed a HIVST delivery strategy that is supported by a risk communication approach.

**Conclusion:**

The NGT enabled successful collaboration with key stakeholders in the co-creation of HIVST delivery strategies to guide implementation and strategy improve urban men’s engagement with HIV services. A follow-up study to evaluate the feasibility of implementing these approaches is recommended.

## Key messages

- The rapid increase in urbanization has led has significantly impacted the epidemiology of infectious diseases such as HIV among men in sub-Saharan Africa.
- Across sub-Saharan Africa, men living with HIV are 20% less likely than women living with HIV to know their HIV status. HIV self-testing (HIVST) presents an opportunity to improve testing rates among this underserved population, however tailored delivery approaches are required.
- Improving access to HIV services for urban men by developing tailored HIVST delivery strategies is key to reducing new HIV infections in high disease burdened settings
- This article presents the most desirable approaches to help improve urban men’s engagement with HIVST

## Background

Across sub-Saharan Africa (SSA), men living with HIV are 20% less likely than women living with HIV to know their HIV status [1, 2]. This presents a major public health problem as knowledge of one’s status is the first and most important step in the HIV care and treatment cascade [3]. Approximately, 7,700,000 people were living with HIV in South Africa and the rate of new infection remains high [4]. South Africa’s HIV programme is the largest in the world and has been making progress towards achieving the Joint United Nations Programme on HIV/AIDS 95–95–95 targets (95% living with HIV know their status, 95% of these on antiretroviral treatment, and 95% with undetectable viral loads) [5]. By end 2020, South Africa’s progress towards the 95–95–95 targets was 92–75–92 [6]. However, most of the gains in achieving the targets have occurred in females living with HIV, while gains in males living with HIV have been modest [5]. There are substantial gaps in HIV service use and coverage for men and boys in South Africa [7]. There is evidence to suggest that, overall, men have lower levels of engagement and retention in HIV care [8, 9] and higher mortality on ART than women [10]. The observed differences in mortalities may be best explained by background differences in mortality between men and women may be related to other factors including men’s poor usage of facility-based health services. In this study, health services is defined as a public service providing HIV testing and treatment services.

To reach the urban men in South Africa efforts to develop HIVST delivery strategies that are tailored to help improve men’s engagement with HIV services urgently needed. In this study, we define HIVST delivery strategies to include all enabling technologies as well all strategies focused on addressing barriers and challenges that are preventing men’s engagement with HIV services. Community-based HIV testing had a significant effect on reaching a high number of HIV-positive men when compared to facility-based testing [11]. HIVST is one of the WHO recommended approaches for HIV testing services [12], particularly for helping reach populations who would not normally access facility-based HIV testing. For larger-scale distribution of community-based interventions such as HIVST (HIVST) to be effective, there is a need to understand the processes required to implement the intervention consistently and at a high level of quality, especially implementing the intervention in different contexts [13]. HIV self-tests are available to the public in South Africa and they can be purchased via pharmacy outlets. HIVST provides a novel and currently severely underutilized supplement to facility-based testing [14]. Oral HIVST offers the potential for increased HIV testing uptake and greater convenience and privacy as well as the potential to increase the proportion of the population who test regularly [14].

Evidence on the acceptability of HIVST in sub-Saharan Africa has suggested higher acceptability of HIVST among men than women [15]. In general, self-testing offers people a self-management solution—empowers people to be in control of their care. In terms of HIV, a highly stigmatized condition, there are many effective ways to deliver and support HIVST (HIVST), depending on the population and setting [16]. Urbanization is one of the fast-growing global trends of the twenty first century. Approximately, 2.5 billion more people will be added to the urban population by 2050, mainly in Africa and Asia [17]. Urbanization can result in a significant impact on individual quality of life, while straining public health systems and resources [17, 18]. The rapid increase in urbanization in SSA having a significantly impact on HIV-related pandemics with men bearing the biggest brunt [19, 20].

There is limited evidence on the most appropriate delivery strategies for HIVST to help improve access to HIV services for urban men in South Africa. HIVST has recently been introduced as a supplementary HIV testing strategy in South Africa and recommended to reach the key and under-tested populations [21]. Therefore, it is important to use appropriate delivery strategies to maximize uptake and impact. It is also recommended that relevant stakeholders are involved in developing and adapting HIVST models. The main objective of this study was to collaborate with stakeholders in the development of HIVST delivery approaches to help improve urban men’s engagement with HIV services. It is anticipated that the results of this study will help guide a planned intervention study to determine the most acceptable delivery strategy for urban men.

## Methods

We invited key stakeholders for urban men’s HIV services such as health care providers, health care service users, government employees, NGOs, and academics to participate in a co-creation workshop aimed at developing HIVST delivery approaches for urban men, using eThekwini municipality as a study setting. We purposely sampled our participants using the snowball sampling or chain-referral sampling techniques. We defined stakeholders as people who have expert knowledge on HIV services, men’s health services, and have an interest in the implementation of HIVST for men in KwaZulu-Natal, South Africa.

### Study participants and sampling

We sent invitation letters (via email) to key stakeholders of HIV services in eThekwini District, non-government organizations (NGOs) involved in men’s health and HIV health services, community representatives, MSM and living with HIV will be recruited through slow balling.

#### Eligibility criteria

We included individuals who meet one of the following inclusion criteria:

- Personnel employed by eThekwini District and responsible for HIV services;
- Personnel employed by eThekwini District or SA-based men’s health and HIV services NGOs
- Men living, working, and studying in eThekwini District for more than 3 months and over the age of 18
- MSM living, working, and studying in eThekwini District and over the age of 18
- Urban men who are living with HIV and over the age of 18
- Individuals who are able to communicate in English language

#### Exclusion criteria

- Personnel who are not involved with HIV testing services in eThekwini District
- People who lack mental capacity to give consent to participate in the study

### Workshop program

During our first engagement with key stakeholders on the 12th of December 2019, we employed the nominal group technique (NGT) method for data collection [22]. The NGT enabled problem identification, solution generation, and decision making among stakeholders. We conducted the workshop in two consecutive phases: phase 1 was focused on determining barriers for men’s engagement with the current/facility-based HIV testing services; phase 2 was aimed at determining HIVST delivery strategies to help improve urban men’s engagement with HIV services. TPM-T and RL facilitated the workshop. The questions asked to the participants at each phase were as follows:

- Phase 1: What are the barriers for men’s engagement with the current/facility-based HIV testing services?
- Phase 2: What are the HIV self-testing delivery strategies that can help improve urban men’s engagement with HIV services?
- Phase 1: We requested key stakeholders to share their knowledge on barriers to urban men’s engagement with current (facility-based) HIV testing services. Following instructions from the facilitator, stakeholders independently grouped their suggestions into themes. The PI (TPM-T) listed the themes in a voting form/questionnaire to enable voting through ranking. Participants were requested to rank the themes according to the level of severity in preventing men’s engagement with current (facility-based) HIV testing and treatment services. The ranking score was between 1 and 5.
- Phase 2: Key stakeholders were requested to propose potential HIVST delivery strategies to help improve urban men’s engagement with HIVST and to group them according to themes, without the PI’s assistance. The PI listed the themes in a voting form/questionnaire to enable voting through ranking. Participants were requested to rank the themes according to the level of effectiveness to enable the delivery of HIVST to urban men. The ranking score was between 1 and 5, 1 being least effective and 5 being the most effective strategy.

Following the NGT workshop, a report presenting the results of NGT was put together by the TPMT and shared with key stakeholders for comments.

### Data management and analysis

To calculate the quantitative data gathered during the ranking step in the nominal group process; for phase 1, a total importance score for each barrier was calculated by summing the individual scores of the participants, for phase 2, a total importance score for each strategy was calculated to denote perceived effectiveness to help address the barriers in phase 1 and improve urban men’s engagement with HIV services. The ranking scores were between 1 and 5, one being the least severe, and five being the most severe barrier to urban men accessing HIVST. The qualitative data were analyzed using thematic content analysis to inductively identify the themes that emerged from the data presented during the discussion. NVivo software was employed for analysis of qualitative data. Two independent coders (TPM-T and an outsourced coder PV) coded the qualitative data with guidance from the main research question of this study. The coding categories were derived directly from the text data to limit researcher biases due to preconceived ideas or other theoretical perspectives.

## Results

Eleven HIV key stakeholders aged 18–60 representing different population groups agreed to participate in our workshop. Of these, 73% were male. The majority (64%) of the study participants were employed, two were unemployed and two were full-time students. Characteristics of participants are presented in Table 1 below.

**Table 1:** Characteristics of workshop participants

Gender	Age group (years)	Occupation
Male	56–65	Unemployed
Male	26–40	Health data specialist (KZN Department of Health)
Male	26–40	High school teacher (KZN Department of Education)
Female	41–55	Postdoctoral student-researching patient-centeredness of HIV services
Male	26–40	Lecturer
Male	18–25	Full-time undergraduate Student
Male	41–55	Research project director for an international NGO
Male	41–55	HIV services program Manager
Female	26–40	HIV counsellor
Male	56–65	Former piping supervisor, current volunteer HIV mentor and counsellor construction industry employees
Male	18–25	Full-time masters student

**Fig. 1:**
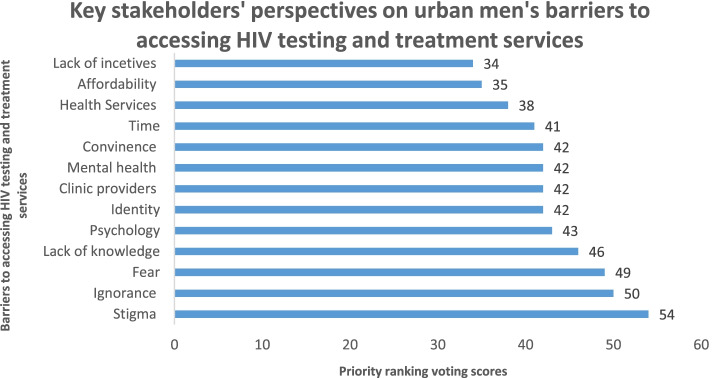
Key stakeholders’ voting scores for urban men’s barriers to accessing HIV self-testing. Concerns relating to scaling up of HIVST

Stakeholders reported thirteen factors as urban men’s barriers to accessing facility-based HIV testing services (Fig. 1). The voting results showed that stigma as the most highly ranked barrier (55 scores) followed by ignorance (50 scores), fear, (49 scores) lack of knowledge on the use of HIVST kits (46 scores), and psychology (43 scores). Lack of incentives (34 scores) was voted as the least severe barrier followed by affordability (35 scores) and health services (38 scores). Psychology was defined as mental characteristics or attitudes that influence an individual’s decision to access facility-based HIV services. Health services referred to structural barriers within a health facility or experienced during health service delivery.

The majority of participating key stakeholders supported the scaling up of HIV self-testing to underserved populations. It is worth noting that some of the stakeholders did not support the scaling up of HIVST to these populations and they raised some concerns. These stakeholders were concerned about HIVST data availability, adequacy of pre- and post-testing counselling, and patients’ reactions to positive test results (Table 2).

**Table 2:** Concerns related to scaling-up of HIVST

Concern	Key stakeholder’ perspectives
Data availability	*‘The major issue is that usage of the HIV test kits as we cannot have proof of the results. All depend on the client to say whether or not they have used it. No data on how many negative or positive clients use tested and how many confirmed test results. It is hard to get this information, except to say how many self-screening kits were distributed. This is similar to condoms, as we see the number of sexually transmitted diseases increase while the number of condom distribution increases.’*
Adequacy of pre- and post-testing counselling	*‘We can broadcast a lot of places and strategies for men to access HIVST but what happens when a person is tested without proper counselling, I think we should introduce a lot of information before scaling up HIVST.’*
Patients’ reactions to positive test results	*‘I don't believe that self-testing will work because we are going to experience more suicides. There will also be an issue with anger, if he finds out on his own and this could lead to more cases of rape and abuse. This will be the start of a fire that will not be stopped. It’s dangerous, I am speaking from a point of view as I am 12 years diagnosed HIV positive and I deal with HIV positive men and women on a daily basis.’*

### HIV testing delivery strategies to improve urban men’s engagement with HIV testing services

A large proportion (73%) of the participants supported scaling up HIVST to help improve urban men’s engagement with HIV testing and treatment services. All 11 participating stakeholders were requested to suggest HIVST delivery strategies and rank them according to their potential effectiveness to help address the above challenges and improve urban men’s engagement with HIV services. Table 3 shows 18 suggested HIVST strategies in ascending order of their ranking score. Key stakeholders ranked the promotion of HIVST via TV adverts as the most desirable (85%) strategy to help enable uptake HIVST and enable urban men’s engagement with HIV testing and treatment services, followed by the use of videos for HIVST pre- and post-testing counselling (80%), promotion of self-testing via social media (80%), placing test kits in pubs (80%), internal motivation strategies (80%), and provision of free testing kits (80%).

**Table 3:** HIV testing delivery strategies to improve urban men’s engagement with HIV testing services

Priority delivery strategies for HIVST	Summing by votes *1 = less effective* *5 = highly effective*					Total number of voting scores (weighted sum = number of votes × ranking score)
	1	2	3	4	5	55
Using celebrities to promote uptake of HIVST	1	1	3	5	1	37
Using endorsement of HIV self-tests by Department of Health	1	1	4	2	3	38
Using sports figures to promote uptake of HIVST		2	3	4	2	39
Providing incentives		2	4	1	4	40
Incorporating mHealth within HIVST		1	4	3	3	41
Partner testing	1		4	1	5	42
Giving men a reason to believe in HIV testing		1	4	2	4	42
Packaging HIVST kits with STI (sexually transmitted disease) self-tests		1	3	4	3	42
Packaging HIV self-tests kits with other diseases screening self-tests		2	2	3	4	42
Providing clear instructions		1	2	5	3	43
Placing test kits in community centres	1	1		5	4	43
Provision of free data	1		1	6	3	43
**Provision of free testing kits**	**1**		**2**	**3**	**5**	**44**
**Internal motivation strategies**			**3**	**5**	**3**	**44**
**Placing test kits in pubs**	**1**	**1**		**4**	**5**	**44**
**Promotion of self-testing via social media**	**1**		**1**	**5**	**4**	**44**
**Use of videos for HIVST pre- and post-testing counselling**	**1**			**7**	**3**	**44**
**Promotion of self-testing via TV adverts**	**1**			**4**	**6**	**47**

*HIVST* HIV self-testing, *STI* sexually transmitted infections, *TV* television

### Reported barrier versus proposed strategies

Our results show a relationship between the most important barriers to accessing HIV testing and some of the suggested HIVST strategies by stakeholders. The use of internal motivation strategies has been proposed as a strategy that could help address three of the most important barriers to accessing HIVST by urban men—stigma, fear, and psychology (Table 4). The group defined internal motivation strategies as provision of appropriate messages that build motivation among men to use HIVST kits. These messages will allow men’s desire to engage in a behavior (HIV test) arising from within the individuals because it is naturally satisfying to them.

**Table 4:** Matching the urban men’s barriers to accessing HIV testing services with proposed HIV self-testing delivery strategies

Urban men’s barriers to accessing HIV services	HIVST delivery strategies
**Stigma (negative attitudes and beliefs about people living with HIV)**	Internal motivation strategies
**Lack of knowledge on the use of HIVST**	Use of videos for HIVST pre- and post-testing counselling
**Ignorance**	• Promotion of self-testing via TV adverts • Promotion of self-testing via social media • Incorporating mHealth within HIVST providing clear instructions
**Fear**	• Internal motivation strategies • Partner testing
**Psychology (mental turbulence of any type which distracts or prevent men from interacting with HIV services)**	Internal motivation strategies

*HIVST* HIV self-testing, *TV* television

### Feedback from stakeholders and suggestions of priority delivery strategies

All 11 workshop participants were requested to comment on the proposed approaches to delivering HIVST to urban men. Of these, all read the report and four provided feedback to the report and provided additional suggestions on the implementation of the suggested priority delivery strategies: internal motivation to help encourage; use of videos for HIVST pre- and post-testing counselling; and the promotion of self-testing via social media

### Suggested HIVST priority strategies

Key stakeholder suggested internal motivation as one of the strategies that can be employed to help encourage the use of HIVST:

“It can be applied to men to engage in an activity because they like it, make them feel good and helps them to take ownership of their personal life/health. The Department of health may engage to motivate men via workshops, televisions ads and writings on papers (e.g.: flyers), school visits may be important too since men engage in physical contacts at young age. Internal motivation may be delivered to men in a form of a story (a person who live with a virus may be a good to share experiences), praises, awards/benefits and recognition.”

“The health professionals that do HIV testing should be males.

Males should be given an option to choose the gender they pre-order to test them.

Empathy should be emphasized by the health workers, e.g. I understand your fear, I’ve also tested etc.”

Use of videos for HIVST pre- and post-testing counselling was also suggested as a one of the strategies to help improve the quality of HIVST testing services to men.

“Through YouTube videos, comprehensively holistic information packs, etc.”

“Cinema ads before the movie stars, urban are engaged in social medias such as face book, WhatsApp and tweeter, self-testing videos maybe posted there by the Department of Health and/or other health organisations.”

“Pre and post counselling videos should be available on YouTube as it is the biggest video platform. The videos should also be available on the department of health website. The self-testing kit should have a link where the videos are available.”

“Data or WIFI opportunities must be available to the person who is considering the self-testing. Videos should be graded incorporating. age, religion, culture, background.”

### Promotion of self-testing via social media

“Internet forum, group chats, group page, etc”

“Through men’s health WhatsApp business account. Facebook pages, Twitter accounts that are created for self-testing. Each community should create a WhatsApp group that is specifically focused on HIVST that and encourages men to test.”

“Community mobilisers, mentor self-testers. Video clips of celebrities promoting self-testing.”

“Male circumcision adverts should include the promotion of self-testing. advertisements should be focused and based on “self -testing is 100% private and confidential” as well as emphasizing where they are available be it public health facilities to stores. As males we tend to see going to the hospital/clinics as something taboo, therefore it will be much more convenient if self-testing kits are not only available in hospitals and clinics.”

“Use men from various background so individuals feel they are represented and can identify with the person/s in the adverts.”

Additional suggestions were provided as part of the feedback to the workshop report to help ensure implementation HIVST to urban men.

“Stickers promoting self-testing may be pasted on cars, taxis and trains. Self/ testing kits should available in local stores such as clicks, medirite, pharmacies, Shoprite, spar etc.”

“Kits should not be expensive so that even unemployed males, pupils and students can access them. Self-testing kits should also be available in universities and schools.”

## Discussion

A collaboration with key stakeholders has enabled collective identification of the most important barriers to accessing current HIV testing and treatment services by urban men and identification of priority areas to be considered while developing HIVST delivery approaches for urban men. It has also revealed that psychological factors as the most important barriers to accessing HIV testing and treatment services by urban men. These included stigma, ignorance, fear, and lack of knowledge. Stigma and ignorance and fear were reported as the top three barriers to men engagement in HIV testing services in this study. Similar findings were reported in previous studies. An earlier study by Sayles et al. 2009 reported that population groups that suffer high levels of stigma were over four times more likely to report poor access to healthcare serves [23]. Studies show that stigma and ignorance deterred young men in South Africa [24], male sex workers in Nigeria [25] and men who have sex with men (MSM) in Lesotho [26] from seeking an HIV testing services. Similar findings were reported in earlier qualitative studies conducted men in South Africa [27, 28].

The World Health Organization refers to poor uptake of HIV services by key populations such as men as a “blind spot” in the global HIV response [1]. Key stakeholders suggested the following priority areas be considered during the development of delivery approached for HIVST for urban men: promotion of self-testing via TV adverts; use of videos for HIVST pre- and post-testing counselling; placing test kits in pubs; promotion of self-testing via social media; provision of free testing kits; and internal motivation strategies.

The benefits of promoting health interventions using television as a means to ensure mass communication and raise awareness has been well established in other studies. In Botswana and Zambia television adverts improved uptake of condoms [29, 30]. Television adverts also improved uptake of voluntary counseling and testing in Ethiopia [31]. Key stakeholders also underscored the need for videos for pre- and post-test counselling. HIVST implementation with online video pre- and post-test counselling improved HIV testing coverage and repeated HIV testing among Chinese MSM [32]. In Malawi, Zambia, and Zimbabwe placing HIVST kits pubs and beerhalls improved uptake among men. Similar recommendations were made by key stakeholders in our study [33].

SSA as a region remains the fastest growing market for smartphone and internet connectivity. 39% of Africans use mobile internet with an anticipated 10% growth by 2025 [34]. Key stakeholders recommended the promotion of HIVST via social media platforms. This aligns with the findings of a systematic review and meta-analysis that showed social media platforms to improve uptake of HIV services among key populations in LMICs [35]. Given the rapid increase in smartphone access and internet connectivity, social media platforms could be instrumental in improving awareness and ultimately uptake. The provision of free testing kits also emerged as an important component of HIVST delivery strategies that improve uptake. This finding corroborates findings from earlier studies conducted among key stakeholders and health care workers in South Africa [36, 37]. While free provision of test kits it admirable, and likely to result in more coverage, the financial feasibility to support scale-up should also be considered. Key stakeholders in the current study advocated for internal motivation strategies to improve uptake of HIVST. Internal motivation strategies that improve men have been recommended for South African men in an earlier study [38].

The collaboration with stakeholders on co-creation of delivery strategies for HIVST for urban men resulted broad range of scenarios around how to support the delivery of HIVST for urban men in KwaZulu-Natal South Africa and other similar settings. Based on the demographics of the stakeholders that participated in this study, we recognize that urban men are not a homogeneous group and that urban environments also have disparities in socioeconomic status. Therefore, in order to elicit the most preferred HIVST delivery approach for different groups of men in preparation for implementation, we propose to conduct a discrete choice experiment (DCE). Packaging HIVST kits with other diseases screening self-tests is one of the suggested delivery strategies by key stakeholders. Bearing in mind the current pandemic and the level of acceptability and uptake of SARS COV-2 self-testing, we recommend that future HIVST intervention studies that are targeted to men should consider combining packaging of SARS COV-2 and HIVST kits to improve the uptake of HIVST by urban men.

### Strengths and limitations of the study

This study employed an innovative research method (NGT) that to enable a systematic collaboration with a wide range of key stakeholders in the co-creation of HIVST delivery approach for urban men. As a results, views different groups of urban men and those of implementers of men’s health services were represented. This enabled generation of a broad range of ideas to guide the development of an acceptable HIVST delivery to improve men’s engagement with health services. The use NGT limited bias of domination of one cadre/participant over others through ranking. Despite this strength, the study had some limitations. First, a group of key stakeholders that were involved in the co-creation of the HIVST delivery strategies did not include pharmacists. Given that HIVST are currently distributed through pharmacies, particularly in urban South Africa, and pharmacists would likely have valuable insights about HIVST use and distribution. Second, since both the users and stakeholders were in the same room, it may have resulted in users being influenced by the presence of providers and not express their views freely or vice versa. Third, the NGT workshop did not include determination of a level of acceptability of the different proposed HIVST delivery approaches by urban men help prepare for successful implementation.

## Conclusion

This study provides a unique opportunity to collaborate with a vast range of stakeholder in the co-creation of HIVST delivery strategies to help improve men’s uptake of HIV services as part of urban development trends to promote health in South Africa. A follow-up study to determine the most preferred HIVST delivery strategies by deferent population groups of urban men is recommended before implementation.

## Data Availability

The raw data analyzed in this study is available upon reasonable written request submitted to the corresponding author.

## References

[1] UNAIDS (2017). Blind spot - Reaching out to men and boys addressing a blind spot in the response to HIV.

[2] Pascoe L, Peacock D, Stemple L. Reaching men: addressing the blind spot in the HIV response. Int J Mens Soc Commun Health. 2018;1(SP1):e57–70.

[3] El-Sadr WM, Harripersaud K, Rabkin M (2017). Reaching global HIV/AIDS goals: what got us here, won't get us there. PLoS Med.

[4] Marcus Low and Sean MacDonell. Graphs that tell the story of HIV in South Africa’s provinces. Available at: https://www.spotlightnsp.co.za/2019/08/05/graphs-that-tell-the-story-of-hiv-in-south-africas-provinces/. Accessed on: 23 June 2020. 2019.

[5] Launch of the South African National HIV Prevalence, Incidence, Behaviour and Communication Survey, 2017. Available at: http://www.hsrc.ac.za/en/media-briefs/hiv-aids-stis-and-tb/sabssm-launch-2018v2. Accessed on: 12 December 2018 [press release]. 2018.

[6] UNAIDS. South Africa 2020. Available from https://www.unaids.org/en/regionscountries/countries/southafrica. Accessed 14 December 2021. 2020.

[7] Day C, Gray A. Health and Related Indicators. HST South Africa Health Rev. 2017. Available from: https://www.hst.org.za/publications/South%20African%20Health%20Reviews/HST%20SAHR%202017%20Web%20Version.pdf. Accessed 14 Dec 2021.

[8] Dube A, Renju J, Wamoyi J, Hassan F, Seeley J, Chimukuche RS (2021). Consequences of male partner engagement policies on HIV care-seeking in three African countries: findings from the SHAPE UTT study. Global Public Health.

[9] Mbuagbaw L, Hajizadeh A, Wang A, Mertz D, Lawson DO, Smieja M (2020). Overview of systematic reviews on strategies to improve treatment initiation, adherence to antiretroviral therapy and retention in care for people living with HIV: part 1. BMJ Open.

[10] Osler M, Cornell M, Ford N, Hilderbrand K, Goemaere E, Boulle A (2020). Population-wide differentials in HIV service access and outcomes in the Western Cape for men as compared to women, South Africa: 2008 to 2018: a cohort analysis. J Int AIDS Soc.

[11] Hensen B, Taoka S, Lewis JJ, Weiss HA, Hargreaves J (2014). Systematic review of strategies to increase men's HIV-testing in sub-Saharan Africa. AIDS.

[12] WHO (2016). Guidelines on HIV self-testing and partner notification: Supplement to consolidated guidelines on HIV testing services.

[13] Glasgow RE, Lichtenstein E, Marcus AC (2003). Why don't we see more translation of health promotion research to practice? Rethinking the efficacy-to-effectiveness transition. Am J Public Health.

[14] Estem KS, Catania J, Klausner JD (2016). HIV self-testing: a review of current implementation and fidelity. Curr HIV/AIDS Rep.

[15] Harichund C, Moshabela M. Acceptability of HIV self-testing in sub-Saharan Africa: scoping study. AIDS Behav. 2018;22(2):560–8. 10.1007/s10461-017-1848-9 PMC5764831 28699017

[16] Colvin CJ. Strategies for engaging men in HIV services. Lancet HIV. 2019;6(3):e191–e200. 10.1016/S2352-3018(19)30032-3 30777726

[17] Zhang XQ (2016). The trends, promises and challenges of urbanisation in the world. Habit Int.

[18] Blekking J, Waldman K, Tuholske C, Evans T (2020). Formal/informal employment and urban food security in Sub-Saharan Africa. Appl Geogr.

[19] Boyce MR, Katz R, Standley CJ (2019). Risk factors for infectious diseases in urban environments of sub-Saharan Africa: a systematic review and critical appraisal of evidence. Trop Med Infect Dis.

[20] Okello S, Amir A, Bloomfield GS, Kentoffio K, Lugobe HM, Reynolds Z (2020). Prevention of cardiovascular disease among people living with HIV in sub-Saharan Africa. Prog Cardiovasc Dis.

[21] Venter F, Majam M, Jankelowitz L, Adams S, Moorhouse M, Carmona S, et al. South African HIV self-testing policy and guidance considerations. South Afr J HIV Med. 2017;18(1). 10.4102/sajhivmed.v18i1.775 PMC5842980 29568643

[22] Delbecq AL, Van de Ven AH (1971). A group process model for problem identification and program planning. J Appl Behav Sci.

[23] Sayles JN, Wong MD, Kinsler JJ, Martins D, Cunningham WE (2009). The association of stigma with self-reported access to medical care and antiretroviral therapy adherence in persons living with HIV/AIDS. J Gen Intern Med.

[24] Maughan-Brown B, Nyblade L (2014). Different dimensions of HIV-related stigma may have opposite effects on HIV testing: evidence among young men and women in South Africa. AIDS Behav.

[25] Crowell TA, Keshinro B, Baral SD, Schwartz SR, Stahlman S, Nowak RG (2017). Stigma, access to healthcare, and HIV risks among men who sell sex to men in Nigeria. J Int AIDS Soc.

[26] Logie CH, Perez-Brumer A, Mothopeng T, Latif M, Ranotsi A, Baral SD. Conceptualizing LGBT stigma and associated HIV vulnerabilities among LGBT persons in Lesotho. AIDS Behav. 2020;24(12):3462–72. 10.1007/s10461-020-02917-y PMC7222929 32394231

[27] Mambanga P, Sirwali RN, Tshitangano T (2016). Factors contributing to men's reluctance to seek HIV counselling and testing at Primary Health Care facilities in Vhembe District of South Africa. Afr J Prim Health Care Fam Med.

[28] Chikovore J, Gillespie N, McGrath N, Orne-Gliemann J, Zuma T, Group ATS (2016). Men, masculinity, and engagement with treatment as prevention in KwaZulu-Natal, South Africa. AIDS Care.

[29] Mokgetse M, Ramukumba MM (2018). Female condom acceptability and use amongst young women in Botswana. Curationis..

[30] Thankian K, Mwaba S, Jere-Folotiya J, Hapunda G, Menon A (2017). Determinants of condom use among currently married men in Zambia. AFRREV IJAH Int J Arts Hum.

[31] Kabeta T, Belina M, Nigatu M (2020). Hiv voluntary counseling and testing uptake and associated factors among sexually active men in Ethiopia: analysis of the 2016 Ethiopian demographic and health survey data. HIV/AIDS (Auckl).

[32] Chan PS-f, Chidgey A, Lau J, Ip M, Lau JT, Wang Z. (2021). Effectiveness of a novel HIV Self-Testing Service with Online Real-Time Counseling Support (HIVST-online) in increasing HIV testing rate and repeated HIV testing among men who have sex with men in Hong Kong: results of a pilot implementation project. Int J Environ Res Public Health.

[33] Hatzold K, Gudukeya S, Mutseta MN, Chilongosi R, Nalubamba M, Nkhoma C (2019). HIV self-testing: breaking the barriers to uptake of testing among men and adolescents in sub-Saharan Africa, experiences from STAR demonstration projects in Malawi, Zambia and Zimbabwe. J Int AIDS Soc.

[34] GSMA. The Mobile EconomySub-Saharan Africa. 2021. Available via: https://www.gsma.com/mobileeconomy/sub-saharan-africa/. Accessed on 2 May 2021

[35] Cao B, Gupta S, Wang J, Hightow-Weidman LB, Muessig KE, Tang W (2017). Social media interventions to promote HIV testing, linkage, adherence, and retention: systematic review and meta-analysis. J Med Internet Res.

[36] Makusha T, Knight L, Taegtmeyer M, Tulloch O, Davids A, Lim J (2015). HIV self-testing could “revolutionize testing in South Africa, but it has got to be done properly”: perceptions of key stakeholders. PLoS One.

[37] Gumede SD, Sibiya MN (2018). Health care users’ knowledge, attitudes and perceptions of HIV self-testing at selected gateway clinics at eThekwini district, KwaZulu-Natal province, South Africa. SAHARA-J J Soc Aspects HIV/AIDS.

[38] Mathenjwa T, Kim H-Y, Zuma T, Shahmanesh M, Seeley J, Matthews P (2019). Home-based intervention to test and start (HITS) protocol: a cluster-randomized controlled trial to reduce HIV-related mortality in men and HIV incidence in women through increased coverage of HIV treatment. BMC Public Health.

